# Co-Culture of Primary Human Bronchial Epithelial Cells at the Air–Liquid Interface and THP-1 Macrophages to Investigate the Toxicity of Polycyclic Aromatic Hydrocarbons

**DOI:** 10.3390/toxics13121065

**Published:** 2025-12-09

**Authors:** Kyle S. Burns, Audrey G. Biggerstaff, Jamie M. Pennington, Susan C. Tilton

**Affiliations:** 1 Department of Environmental and Molecular Toxicology, Oregon State University, Corvallis, OR 97331, USA; 2 Superfund Research Center, Oregon State University, Corvallis, OR 97331, USA

**Keywords:** co-culture model, in vitro toxicity, polycyclic aromatic hydrocarbons (PAHs), THP-1 cells

## Abstract

The development of new approach methodologies that include human cells differentiated into organotypic formats is of high interest due to their structural and functional similarities to tissues in vivo, enabling mechanistic understanding and translation to adverse health outcomes in humans. However, these systems often fail to capture complex intercellular signaling required for processes such as pulmonary inflammation induced by polycyclic aromatic hydrocarbons (PAHs). To investigate airway epithelial–macrophage interactions in response to benzo[a]pyrene and a PAH mixture (Tox Mix), co-culture models utilizing primary human bronchial epithelial cells (HBECs) differentiated at the air–liquid interface were cultured with THP-1 macrophages either directly or indirectly, alongside HBECs alone. After 24 h of exposure, cytokine expression (*IL1B*, *IL6*, *CXCL8*, *TNF*) as well as PAH biomarkers previously identified for chemical metabolism (*CYP1A1*, *CYP1B1*), oxidative stress (*ALDH3A1*, *HMOX1*, *NQO1*), and barrier integrity (*TJP2*) were evaluated. Cytotoxicity and barrier integrity were also assessed. HBECs alone and direct co-cultures exhibited similar responses after PAH treatment, while indirect co-cultures showed lower sensitivity to induction of inflammatory cytokines and *CYP1A1* and *CYP1B1* biomarker expression following exposure to PAHs. The expression of other biomarkers, including *ALDH3A1*, *HMOX1*, and *NQO1*, remained largely consistent across all models after treatment. Overall, these findings suggest that direct co-culture systems may provide a more physiologically relevant platform for studies of PAH-induced toxicity and demonstrate that the configuration of co-culture systems can influence cellular responses to chemical exposure.

## 1. Introduction

In the respiratory system, induction of airway inflammation is orchestrated through signaling between airway epithelial cells and macrophages, which are two of the most abundant cell types in the respiratory tract contributing to the innate immune response [1]. The epithelium serves as an initial defense against inhaled chemicals, particles, and pathogens both as a physical barrier and as an actor for the immune system. When an environmental insult occurs, epithelial cells can promote an inflammatory response through the release of mediator molecules. Common mediators of inflammatory responses include cytokines such as IL-1β, IL-6, CXCL8, and TNF which can increase the production of reactive oxygen species, induce apoptosis, and recruit and activate other immune cells [2]. This activation of airway immune response and inflammation are critical events that can lead to adverse health effects and damage to the respiratory system after insult from various external agents.

One class of such external agents, polycyclic aromatic hydrocarbons (PAHs), are chemicals consisting of multiple aromatic rings. PAHs can be formed from pyrolytic and incomplete combustion processes such as the burning of brush and trees in a wildfire or the burning of fuel in combustion engines. PAHs are semi-volatile and can be in a vapor phase as well as bound to particulate matter (PM) in the air [3]. Airway inflammation resulting from exposure to PAHs either alone, in environmental mixtures, or in particulate matter (PM) has been observed in epidemiological studies. For example, in a study by Barraza-Villarreal et al. (2014), PAH exposure, as measured by urinary PAH metabolites, showed a negative association with lung function (FEV1 and FVC) and with pH of exhaled breath condensate, which served as a marker of airway inflammation, in Mexican schoolchildren [4]. In another study, Xiao et al. (2023) showed an association between the systemic immune-inflammation index (SII) and 2-hydroxyfluorene, a metabolite of fluorene, indicating that PAHs may be able to trigger a systemic immune response in response to airway inflammation in exposed individuals [5]. PAH exposure has been linked to increased risk of asthma and other obstructive pulmonary diseases due in part to local airway inflammation [6]. Because of the difficulty in parsing the role of PAHs from particulate matter and other pollutants in epidemiological studies, in vitro models serve as a platform to investigate molecular mechanisms in humans. However, many of these studies have used either immortalized cell lines or focused on a single cell type [7,8,9,10,11,12,13,14,15,16], so the signaling mechanisms leading to inflammatory responses following PAH exposure are not understood. Previous studies have demonstrated a dual role of the xenobiotic-sensing aryl hydrocarbon receptor (AhR), a ligand-activated transcription factor that mediates many PAH responses, in controlling inflammatory responses [8]. Additionally, in vitro studies have demonstrated that signaling through immune receptors such as toll-like receptors can sensitize cells to respond to PAHs [7]. Taken together, these studies indicate complex in vivo responses to PAHs.

There is growing interest in developing more human-relevant in vitro models for toxicity testing to improve mechanistic understanding and translation to adverse health outcomes in humans. The development of new approach methodologies (NAMs) that include human cells differentiated into organotypic formats is of high interest due to their structural and functional similarities to tissues in vivo [17,18]. However, many of these models continue to be inadequate for representing cell signaling required for more complex biological processes, such as pulmonary inflammation, which requires communication between multiple cell types. One promising option for investigating respiratory–immune interactions in vitro is in the development of co-culture models that incorporate lung epithelial cells and immune cells. A variety of co-culture formats have been proposed utilizing cell culture inserts, each with their own benefits and limitations; however, models that rely on primary lung cells co-cultured at the air–liquid interface (ALI) in a manner that allows for investigation into the contribution of each cell type are limited [19,20,21]. For example, many models utilize only immortalized cell lines that are not differentiated at the ALI, which limits their similarity to the in vivo lung despite the increasing usage and availability of primary cells [22,23,24,25]. In contrast to cell lines, primary human bronchial epithelial cells (HBECs) differentiated at the ALI retain characteristics similar to in vivo lungs with the formation of a pseudostratified and heterogeneous cell layer consisting of various epithelial cell types that globally resemble in vivo transcription [25,26,27]. Therefore, to investigate the combined contribution of airway epithelial-macrophage response to mechanisms of PAH toxicity in vitro, two different co-culture models were compared, in which primary HBECs differentiated at the ALI (ALI-HBEC) on cell culture inserts were cultured in the presence of THP-1 macrophages in different formats and evaluated for pro-inflammatory cytokine markers, biomarkers of PAH exposure, cytotoxicity, and barrier integrity.

## 2. Materials and Methods

### 2.1. Chemicals and Reagents

Gibco^TM^ Dulbecco’s phosphate-buffered saline without calcium and magnesium ions (DPBS) and trypsin were purchased from Thermo Fisher Scientific (Waltham, MA, USA). SsoAdvanced Universal SYBR Green Supermix was purchased from Bio-Rad Laboratories (Hercules, CA, USA). PneumaCult™-Ex Plus medium and PneumaCult™-ALI medium were purchased from STEMCELL Technologies (Vancouver, BC, Canada). Primary HBECs were purchased from Lonza (Basel, Switzerland). THP-1 cells and RPMI-1640 were purchased from ATCC (Manassas, VA, USA). Benzo[a]pyrene (BAP; CAS# 50-32-8, 96% purity) was purchased from BeanTown Chemical (Hudson, NH, USA). A PAH mixture, Tox Mix, was formulated as previously described [28]. Briefly, Tox Mix is a synthetic mixture derived from environmental passive air sampling at a legacy creosote site concurrently impacted by wildfire smoke. In order of molar abundance, Tox Mix comprises retene (72.8%), benzo[a]fluorene (14.6%), benzo[b]fluorene (7.28%), benzo[c]fluorene (3.64%), triphenylene (1.45%), benzo[e]pyrene (0.145%), and benzo[ghi]perylene (0.091%). Details on each individual standard can be found in Table S1.

### 2.2. Air–Liquid Interface Human Bronchial Epithelial Cell Cultures

Primary human bronchial epithelial cells (HBECs) were cultured as previously detailed [26]. Briefly, passage 2 HBECs were expanded in PneumaCult^TM^ Ex-Plus medium (STEMCELL Technologies, Vancouver, BC, Canada) until ~85% confluent and the cells were harvested by trypsinization. HBECs were then seeded onto 6.5 mm diameter Transwell^®^ inserts with 0.4 μm membrane pores (Corning, Corning, NY, USA), with 500 µL Ex-Plus in the basolateral chamber and 200 µL Ex-Plus in the apical chamber. After HBECs were confluent on inserts, Ex-Plus media was removed from both chambers and 500 µL of PneumaCult^TM^ ALI medium (STEMCELL Technologies, Vancouver, BC, Canada) was added to the basolateral chamber to induce differentiation at the air–liquid interface. Cells were maintained at 37 °C with ALI medium replaced every 48–65 h. After day 14 post-air–liquid interface induction, HBECs were washed with 200 µL DPBS every 7 days to remove mucus.

### 2.3. THP-1 Cell Cultures

THP-1 cells (ATCC, Manassas, VA, USA) were cultured in RPMI-1640 (ATCC, cat #: 30-2001) supplemented with 10% FetalClone II serum (Cytiva, Marlborough, MA, USA). Media were refreshed when cultures reached ~8 × 10^5^ cells/mL with subcultures at ~2 × 10^5^ cells/mL. THP-1 were maintained at 5% CO_2_ and 37 °C.

THP-1 monocytes were differentiated to macrophages with phorbol 12-myristate 13-acetate (PMA; Sigma-Aldrich, St. Louis, MO, USA) at concentrations ranging from 8.1 to 250 nM in 0.5% DMSO with RPMI-1640 medium at a density of 2.5 × 10^5^ cells/mL for 48 h, with 100 nM PMA used for the co-culture experiments.

Following incubation of THP-1 cells with PMA, media were collected and the number of THP-1 cells in suspension were counted on a TC20 Automated Cell Counter (Bio-Rad Laboratories, Hercules, CA, USA). Viability of THP-1 cells in suspension was determined by trypan blue exclusion.

For the direct co-culture model, 0.025% trypsin (Thermo Fisher Scientific, Waltham, MA, USA) was added at 0.08 mL/cm^2^ and incubated at 37 °C for 30 min. The resulting cell suspension was then mixed thoroughly before adding 1 mg/mL trypsin inhibitor (Sigma-Aldrich, St. Louis, MO, USA) at 0.064 mL/cm^2^. This combination of trypsin and inhibitor was used because it was found to minimize aggregation of cells while still harvesting them. For the indirect co-culture model, PMA-containing media were removed and wells were rinsed with DPBS; macrophages were not trypsinized.

### 2.4. HBEC–Macrophage Co-Culture

The direct co-culture was prepared similarly to the methods used by Rothen-Rutishauser et al. (2005) [21]. After obtaining the macrophage solution, inserts with ALI-HBEC at day 47 post-differentiation were inverted and 50 µL of a 2.7 × 10^6^ cells/mL macrophage solution in ALI medium was slowly added. Large 150 × 25 mm cell culture plates (Corning, Corning, NY, USA) containing the inverted inserts were then carefully placed in an incubator at 37 °C with 5% CO_2_ for 2 h to allow the macrophages to adhere to the inserts [21]. At the end of the 2 h incubation, the basolateral side of the inserts was rinsed with DPBS to remove suspended THP-1 and the inserts were returned to fresh ALI media.

For the indirect co-culture model, 500 µL ALI medium was added to each well containing THP-1 macrophages following THP-1 differentiation. Transwell^®^ inserts containing ALI-HBEC on day 49 post-differentiation were then transferred to wells containing THP-1 macrophages. A diagram showing a brief overview of each co-culture model setup can be found in the Supplementary Materials (Figure S1).

### 2.5. Chemical Exposures

Chemicals were prepared in dimethyl sulfoxide (DMSO), then diluted to 1% DMSO (*v*/*v*) in DPBS for exposure. Prior to exposure, the apical side of inserts containing HBECs were washed 3 times with 200 µL DPBS to remove mucus. The apical surface was then treated for 24 h with 25 µL vehicle control (1% DMSO *v*/*v* in DPBS), Tox Mix (55 and 277 µM), or BAP (159 µM). This dosing was equivalent to 0.96 µg/cm^2^ Tox Mix, 4.82 µg/cm^2^ Tox Mix, and 3.04 µg/cm^2^ BAP on the surface of cells, respectively.

### 2.6. Transepithelial Electrical Resistance Measurement

Transepithelial electrical resistance (TEER) was measured prior to the chemical exposures and at the end of the 24 h exposure period. TEER was measured via an EVOM2 epithelial voltohmmeter with an STX2 electrode (World Precision Instruments, Sarasota, FL, USA). Before measuring TEER across Transwell^®^ inserts, the voltohmmeter was calibrated using a 1 k-Ω test resistor and dipped in isopropyl alcohol. The probe was placed in DPBS when not in use. Mucus was washed from the apical side of inserts 3 times with 200 µL DPBS before an additional 200 µL DPBS was added to the apical chamber and 500 µL DPBS added to the basolateral chamber for measuring TEER. For the direct co-culture, electrical resistance across Transwell^®^ inserts with only THP-1 macrophages was measured but was found to result in values equivalent to an empty insert.

### 2.7. Lactate Dehydrogenase Cytotoxicity Assay

Lactate dehydrogenase (LDH) activity in media was measured using a CyQUANT^TM^ LDH Cytotoxicity Assay Kit (Invitrogen, Thermo Fisher Scientific, Waltham, MA, USA) to evaluate relative cytotoxicity following exposure to PAH mixtures or vehicle control. The manufacturer’s instructions were followed in performing this assay. Briefly, 50 µL of co-culture media was combined with 50 µL of the reaction mixture and incubated in the dark at room temperature for 30 min. After 30 min, 50 µL of stop solution was added and mixed. Absorbance was read at 490 nm and 680 nm using a Synergy HTX plate reader (BioTek, Winooski, VT, USA), and LDH activity was determined by subtracting A_680_ from A_490_.

### 2.8. Evaluation of Gene Expression by qPCR

HBECs were scraped, collected in DPBS, and stored at −80 °C. Cells were centrifuged and DPBS was removed before total RNA was extracted and isolated from cells using an RNeasy Mini kit (Qiagen, Hilden, Germany) following the manufacturer’s protocol. RNA was then evaluated for purity and quantity via Take3 module on a BioTek Synergy HTX plate reader (Agilent Technologies, Santa Clara, CA, USA).

To generate cDNA from total RNA, iScript™ Reverse Transcription Supermix for RT-qPCR kits (Bio-Rad Laboratories) were used in an Applied Biosystems 2720 Thermal Cycler (Thermo Fisher). Quantitative PCR (qPCR) reactions were run by combining 2 µL of cDNA, 150 nM of each forward and reverse primer, 5 µL of SSoAdvanced Universal SYBR Green Supermix (Bio-Rad Laboratories), and nuclease-free water in a CFX96 Touch Real-Time PCR Detection System (Bio-Rad Laboratories). The CFX96 thermocycler used the following program: 1 cycle at 95 °C for 1 min (initial denaturing), 40 cycles at 95 °C for 15 s (denaturing), 60 °C for 30 s (annealing/elongation), and a melt curve of 65–95 °C/0.5° per 5 s. Differences in relative expression among treatments compared to the vehicle control were then calculated using the ΔΔCt comparative method and normalized to a housekeeping gene. Peptidylprolyl isomerase A (*PPIA*) and β-actin (*ACTB*) were used as housekeeping genes for HBEC and THP-1 RNA, respectively. Primer sequences can be found in Table S2.

### 2.9. Statistical Analysis

After collection, data were normalized to vehicle control and outliers were identified by Grubbs’ Test (α = 0.05). Data collected from PMA treatments with more than three doses were analyzed by one-way ANOVA followed by Dunnett’s post hoc test. Where applicable, a one-way ANOVA followed by Tukey’s post hoc test was used for THP-1 experiments with three treatment groups and for comparing vehicle responses across the three models with HBEC. LDH, TEER, and gene expression data were normalized to the respective model vehicle control. A two-way ANOVA (Type III) was then performed to allow for determination of model–treatment interaction followed by Tukey’s post hoc test for main effects (model and treatment). For the ANOVAs and t-tests, a *p*-value below 0.05 was considered to be statistically significant, whereas an adjusted *p*-value of less than 0.05 was considered to be statistically significant for post hoc tests. For further investigation into each model’s response to vehicle treatment, vehicle treatment groups are shown normalized to the HBEC-alone vehicle in separate plots.

## 3. Results and Discussion

### 3.1. Importance of Co-Culture Models for Respiratory Toxicity Testing

Despite the frequent use of animal models for respiratory toxicology studies, there are a number of differences between human airways and those of other animals, including differences in cytochrome P450 (CYP) metabolic profiles among organisms [29]. This is particularly relevant to PAH toxicity since parent PAHs are not generally genotoxic until metabolized and could lead to discrepancies in PAH toxicity between species [29,30]. Because of these differences, an in vitro model that better recapitulates the human airway is necessary for toxicological studies to ensure accurate evaluations of chemical hazards. To address this need, in vitro models with increased physiological relevance such as metabolically competent primary ALI-HBEC models have been utilized [31,32]; however, many of these models continue to be inadequate for representing immune responses that require signaling between multiple cell types.

Co-culture systems are a promising option for investigating respiratory–immune interactions in vitro and present an opportunity to better recapitulate in vivo response than models consisting of a single cell type [33]. A variety of different co-culture models with various combinations of cell types have been utilized in previous studies: epithelial–endothelial, epithelial–immune, epithelial–endothelial–immune, etc. [34,35]. This added complexity enables more accurate determination of human health hazards in vitro compared to models comprising a single cell type. With regard to epithelial–immune co-cultures, macrophages and epithelial cells are two of the most abundant cell types in the respiratory tract and are thought to orchestrate airway inflammation [1], so addition of macrophages to ALI-HBEC could be informative for evaluating chemicals for inflammatory responses in vitro.

Many studies have performed comparisons between cell culture models with various combinations of cell types [36,37,38]; however, relatively few studies have compared between co-culture models with differences in cell type location or co-culture setup of the same cell types. In the case of location, cell types cultured directly may have contact interactions not present if the cells are separated as well as increased localized concentrations of signaling molecules. To address this, we proposed the use of two different co-culture models utilizing HBECs and THP-1 macrophages. In both models, HBECs differentiated at the ALI were cultured on Transwell^®^ inserts before being combined with THP-1 macrophages. In the indirect co-culture, macrophages were located on the bottom of culture plate wells, whereas in the direct co-culture, macrophages were seeded onto the basolateral side of inverted Transwell^®^ inserts. Supplemental Figure S1 diagrams these models.

### 3.2. Confirmation of THP-1 Differentiation and Identifying Optimal PMA Concentration

Prior to co-culturing THP-1 macrophages and HBECs, it was necessary to identify suitable conditions for co-culturing due to lack of a standardized protocol for THP-1 differentiation for a co-culture model system utilizing primary HBECs at the ALI [23,39,40,41]. The methods used to differentiate THP-1 monocytes into macrophages vary by chemical stimulus (phorbol-12-myristate-13-acetate, vitamin D3, retinoic acid) [42,43,44,45], incubation time with the stimulus (ranging from 3 h to 72 h) [42,43], and exclusion or inclusion of a rest period (ranging from a couple hours to over a week) [42,43,45,46]. While some studies have used additional stimuli to polarize THP-1 macrophages to a specific phenotype [47], it is important to note that all of these aforementioned factors affect the resulting phenotype [42,43,48]. Of note, higher concentrations of PMA have been shown to produce a more activated, pro-inflammatory THP-1 macrophage phenotype than lower PMA concentrations [42,43]. Because the resulting phenotype of THP-1 cells depends upon the method used for differentiation and there is no standardized method for differentiation [42], comparisons between studies utilizing THP-1 macrophages is challenging. In the case of co-culture models, comparisons across studies become even more challenging, since combining the number of co-culture formats with the variety of THP-1 differentiation methods results in a large number of potential co-culture models. There is even evidence that THP-1 macrophage phenotype may be influenced by contact with HBECs [49].

To assess culture parameters and optimize conditions necessary for co-culture, two co-culture methods were compared in which THP-1 macrophages were either seeded onto the inverted Transwell^®^ insert (direct model) or onto the bottom of a cell culture plate well (indirect model). Both models utilized primary HBECs, containing a variety of epithelial subtypes, on the apical side of the Transwell^®^ insert and THP-1 macrophages in the basolateral chamber. HBEC and THP-1 cells were cultured on opposite sides of the Transwell^®^ membrane to simplify analysis and determination of how each cell type (HBEC or macrophage) contributed to the response to PAH exposure. While the setup involved for the indirect model was simpler compared to that of the direct model, there was increased distance between HBECs and THP-1 macrophages in the indirect model, which we hypothesize impacts signaling between the two compartments. Additionally, the setup of each model differed in that the density of macrophages (cells/cm^2^) was lower in the indirect co-culture compared to the direct co-culture despite the total number of macrophages being roughly the same between models.

**Figure 1:**
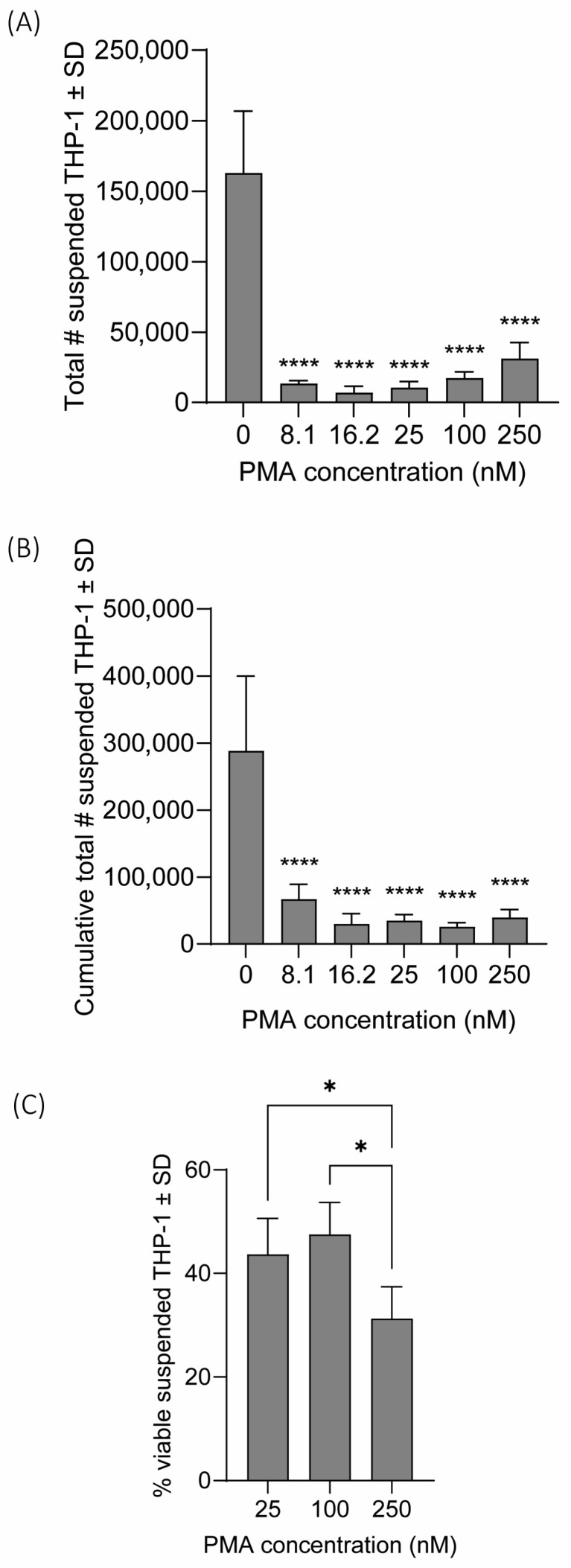
Suspended THP-1 cell counts and viability. (**A**) THP-1 cells were treated with PMA (0–250 nM) for 48 h before culture media was removed and THP-1 cells in suspension were counted. (**B**) Fresh RPMI-1640 was added after the 48 h incubation with PMA and THP-1 cells had a ‘rest’ period for 24 h before THP-1 cells in suspension were counted again. (**C**) Viability of suspended THP-1 cells after 48 h PMA incubation was determined by trypan blue exclusion. Statistical significance is indicated by asterisks. For cell counts, data was analyzed by one-way ANOVA followed by Dunnett’s post hoc test (* *p*-adj < 0.05, **** *p*-adj < 0.0001). For viability, data was analyzed by one-way ANOVA followed by Tukey’s post hoc test (* *p* < 0.05, **** *p* < 0.0001).

Following incubation of THP-1 cells with PMA for 48 h, the number of THP-1 cells in suspension were counted to identify a concentration of PMA with minimal suspended THP-1 cells. For all concentrations of PMA (8.1–250 nM), there was a significant decrease in the amount of THP-1 in suspension after incubating with PMA for 48 h compared to the 0 nM control (*p*-adj < 0.0001 for all; Figure 1A). The lowest amount of THP-1 in suspension resulted from 16.2 nM PMA, with a positive association between the amount of THP-1 in suspension and PMA concentration for concentrations greater than 16.2 nM. However, since THP-1 macrophages would be co-cultured with HBECs for 24 h after seeding, we evaluated the cumulative amount of THP-1 in suspension by combining the amount of THP-1 in suspension following 48 h incubation with PMA and 24 h after seeding macrophages. This resulted in 100 nM having the lowest mean value of THP-1 in suspension (Figure 1B). We also evaluated the viability of suspended THP-1 cells following the 48 h incubation with PMA by trypan blue exclusion. There was no statistically significant difference in viability between 25 and 100 nM (*p*-adj > 0.05), but there was a significant difference between 100 and 250 nM (*p*-adj < 0.05; Figure 1C), so we opted for 100 nM PMA for use in future co-culture experiments. It is important to note that viability of THP-1 in suspension likely does not reflect the viability of adhered macrophages since apoptotic macrophages could detach and skew the percentage of viable suspended cells [50].

**Figure 2:**
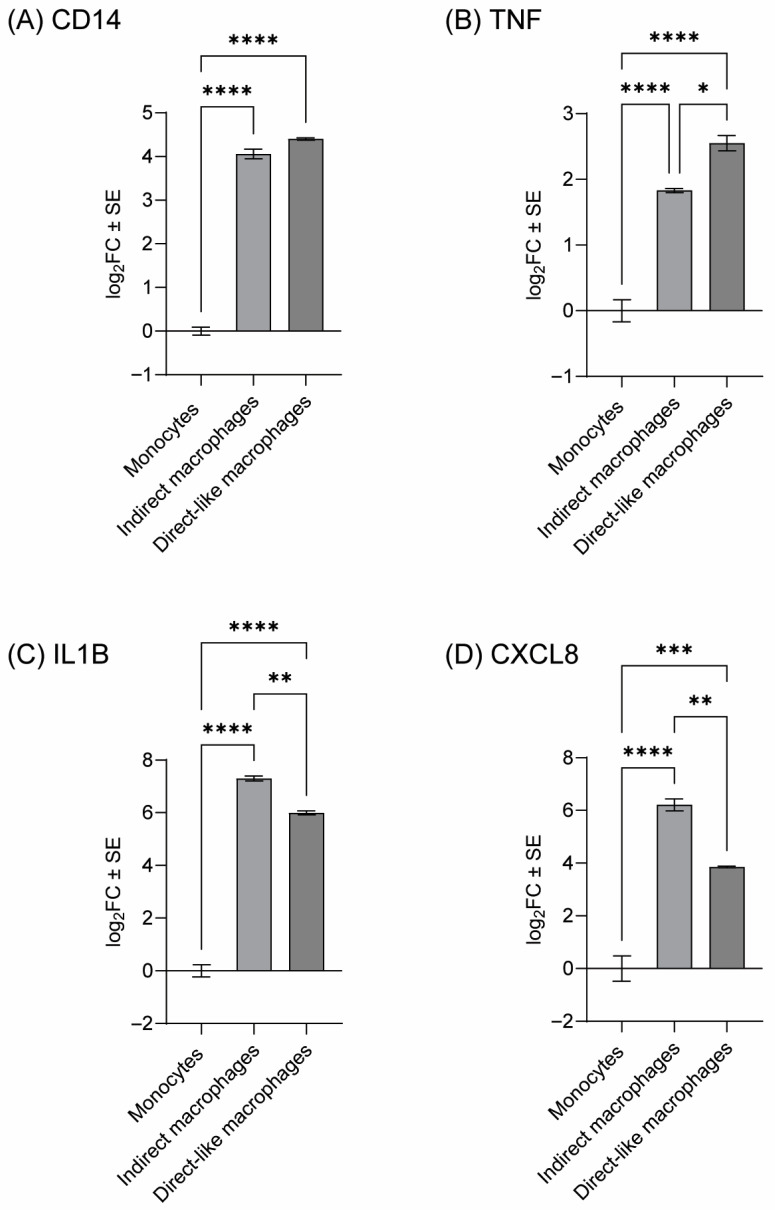
Gene expression in THP-1 cells. THP-1 cells were differentiated into macrophages by following either the indirect or direct co-culture methods using 100 nM PMA, and THP-1 cells were treated in parallel with vehicle control (0.5% DMSO in RPMI-1640). Gene expression was then evaluated by qPCR for (**A**) *CD14*, (**B**) *TNF*, (**C**) *IL1B*, and (**D**) *CXCL8*. Data points represent the mean log_2_ fold change (log_2_FC) in gene expression compared to the monocytes. Error bars represent the standard error of the means. Statistical significance is indicated by asterisks (* *p* < 0.05, ** *p* < 0.01, *** *p* < 0.001, **** *p* < 0.0001; one-way ANOVA followed by Tukey’s post hoc test).

To further confirm differentiation of THP-1 monocytes into macrophages, we evaluated changes in gene expression of a macrophage marker (*CD14*) and cytokine markers (*IL1B*, *CXCL8*, and *TNF*) in THP-1 following incubation with or without PMA and following a similar setup method to either indirect or direct co-culturing [43]. Both setup methods resulted in statistically significant increases (*p*-adj < 0.001) in expression of all four markers compared to monocytes (Figure 2A–D). For three of the four genes (*IL1B*, *CXCL8*, and *TNF*), there were also statistically significant differences between macrophage groups with the direct setup resulting in a greater increase in *TNF* (Figure 2B), and the indirect setup resulting in a greater increase in *IL1B* and *CXCL8* (Figure 2C,D).

THP-1 macrophages used in direct co-culture were also trypsinized prior to co-culturing whereas macrophages in indirect co-culture were not—a difference that was intended to simplify the setup of the indirect co-culture. Trypsin has been shown to increase TNF protein expression in THP-1 which is in agreement with the increase in TNF mRNA expression observed in the macrophages trypsinized for the direct model compared to the macrophages not trypsinized for the indirect model (Figure 2B) [51]. PAR2, a protease-activated receptor that plays a role in inflammation and the immune response, is activated by trypsin and can lead to an induction of *TNF*. Therefore, PAR2 activation may contribute to the response of TNF in THP-1 macrophages in direct co-culture.

Overall, there are many factors that need to be considered to develop an epithelial–immune co-culture model. These factors include cell types to be used, location of each cell type in the system, and choice of medium, to name a few. In the case of differentiating monocytes into macrophages, there are further considerations such as differentiating stimulus, incubation time with the stimulus, inclusion of a rest period, impact of detachment methods on macrophages, etc. [52]. All these factors need to be considered based on the intended use of the co-culture system and must be evaluated to fully validate the system; therefore, the systems utilized have not been fully validated and future studies may be beneficial for further optimization of the co-culture models.

### 3.3. Barrier Integrity and Cytotoxicity

In the respiratory system, the epithelium serves as an initial defense against inhaled chemicals, particles, and pathogens as a physical barrier and orchestrates induction of airway inflammation in conjunction with macrophages [1]. In response to inflammatory cytokines, the epithelial barrier can be weakened or damaged, as is seen in models of asthma and other chronic respiratory diseases; however, the reverse is also true, where damage to the epithelial barrier can trigger an inflammatory response [1,26,53]. Ultimately, a reduction in epithelial barrier integrity and increased epithelial permeability can result in increased uptake of environmental agents such as toxicants and pathogens.

Epithelial airway cells alone have the capacity to release mediator molecules such as TNF in response to toxicant exposure which can result in altered barrier integrity and/or cytotoxicity; however, macrophages also can release signaling molecules that initiate similar effects in epithelial cells [2,53,54]. Therefore, an in vitro model utilizing only epithelial cells may not accurately reflect changes in barrier integrity and/or cytotoxicity in response to PAH exposure.

**Figure 3:**
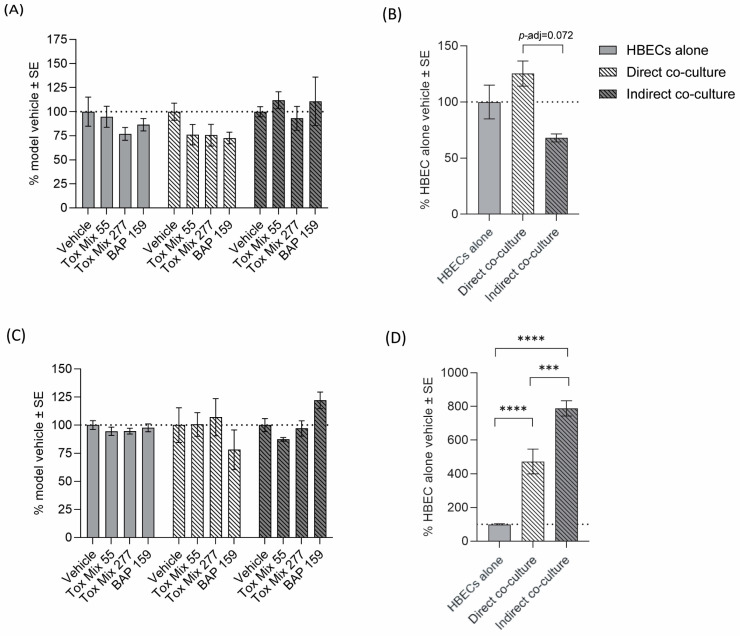
Barrier integrity and cytotoxicity. Cultures of HBEC alone, direct co-culture, and indirect co-culture were exposed to vehicle, Tox Mix, or BAP in 1% DMSO vehicle control. (**A**,**C**) Barrier integrity was then evaluated by measuring transepithelial electrical resistance (TEER). (**B**,**D**) Cytotoxicity was determined by measuring lactate dehydrogenase (LDH) activity in basolateral media. For visualization of treatment effects, data has been normalized to the respective model vehicle (**A**,**C**). Comparisons between vehicle treatments are shown as normalized to the HBEC-alone vehicle (**B**,**D**). Data points represent the mean response in each group compared to the vehicle control presented as the % change normalized to vehicle control. Error bars represent the standard error of the means. Statistical significance is indicated by asterisks *** *p*-adj < 0.001, **** *p*-adj < 0.0001; two-way ANOVA followed by Tukey’s post hoc test).

To evaluate the barrier integrity of the HBEC layer, we measured transepithelial electrical resistance (TEER) following chemical exposure. A blank control was also measured, and the value was subtracted from sample values before normalizing the data as discussed in the Methods and Materials. The two-way ANOVA resulted in no statistical significance for differences between treatments or interaction between model and treatment (*p* > 0.05; Figure 3A). While not statistically significant, the indirect co-culture model was the least impacted by chemical exposure when compared to its vehicle control. In prior studies using HBEC alone, a reduction in barrier integrity has been observed after treatment with some PAHs and mixtures, including Tox Mix, after 24–48 h exposure depending on treatment or timepoint [10,26,55]. In the present study, there was no statistically significant change in barrier integrity, but a decrease in TEER values was observed in response to BAP and Tox Mix in the HBEC-alone model, consistent with prior studies.

Like barrier integrity, cytotoxicity can also be a result of increased inflammation or trigger an inflammatory response [56]. To evaluate cytotoxicity in response to PAH exposure across the different cell models, we measured activity of lactate dehydrogenase (LDH) released into the cell media after 24 h of exposure. Treatment alone did not result in cytotoxicity across models (Figure 3B). Prior studies using HBEC alone, where cells were exposed to increasing concentrations of BAP or Tox Mix for 24–48 h, also did not result in cytotoxicity under most conditions; although, significant cytotoxicity less than 20% of control was observed with BAP at higher concentrations after 48 h and may be related to formation of metabolites [10,26,32,55].

Absent of PAH treatment, there was not a statistically significant difference in barrier integrity between models within the vehicle-treated group (Figure 3C); however, the direct co-culture had relatively increased TEER values compared to the indirect co-culture (*p*-adj = 0.072). As for cytotoxicity, both co-culture models had increased release of LDH into media compared to HBEC alone (*p*-adj < 0.0001), with the indirect co-culture also showing greater cytotoxicity compared to the direct co-culture (*p*-adj < 0.0004; Figure 3D). This increase in relative LDH in media in both co-culture models compared to HBEC alone was expected due to the greater overall number of cells in the co-culture model systems.

Previous studies have shown an increased cytotoxic response in co-culture compared to models consisting of a single cell type. While no significant cytotoxicity was observed relative to controls, Melzi et al. (2024) reported increased cytotoxicity in a Calu-3 and THP-1 macrophage co-culture compared to Calu-3 cells alone following exposure to particulate matter (PM) generated from liquid fuel in a Combustion Aerosol Standard generator [57]. However, this difference was not observed following exposure to PM generated from a different method and may be a result of differences in mixture composition. As another example of differences between monocultures and co-cultures, Zhou et al. (2022) reported that BEAS-2B cells cultured with U937 macrophages were less sensitive to PM_2.5_, nitrosamine ketone, and benzo(a)pyrene diol epoxide than BEAS-2B cells alone in various endpoints including cell proliferation, apoptosis, and DNA damage [58]. Notably, their co-culture system more closely resembles the indirect co-culture described in our study, and we generally observed reduced sensitivity across all endpoints assessed in indirect co-culture compared to HBEC alone. Wang et al. (2020) demonstrated greater sensitivity to PM in a submerged co-culture compared to an ALI co-culture consisting of A549 and THP-1 macrophage cells [23]; however, they hypothesized that the ALI model demonstrated reduced sensitivity due to the production of surfactant and likely better represented physiological conditions.

### 3.4. Cytokine Gene Expression in HBEC

To investigate potential pro-inflammatory effects of PAH exposure, changes in gene expression were evaluated for four pro-inflammatory cytokines—*IL1B*, *IL6*, *CXCL8*, and *TNF*—in HBECs in the presence or absence of macrophages. Epithelial cells and macrophages can promote an inflammatory response through the release of cytokines in response to environmental insult. Through these molecules, alongside danger-associated molecular patterns (DAMPs), macrophages can induce specific responses in epithelial cells to promote an inflammatory response in the epithelium such as induction of the aforementioned cytokines and address the foreign chemical, pathogen, or particles [59].

Cultures of either HBECs alone or HBECs with macrophages in either direct or indirect co-culture models were exposed to PAH solutions by dosing the apical side of inserts and RNA was then isolated from HBECs. One benefit of the co-culture models presented in this work is the ease of isolating HBECs separately from THP-1 cells. Other laboratories have utilized co-culture models where epithelial cells are cultured in the same chamber as macrophage cells [35,60]. While these co-culture models enable increased interaction between the cell types, there is a challenge in determining the contribution of each cell type to the overall observed response. Here, we harvested RNA from HBECs isolated from indirect and direct co-culture models to evaluate the response of epithelial cells specifically.

**Figure 4:**
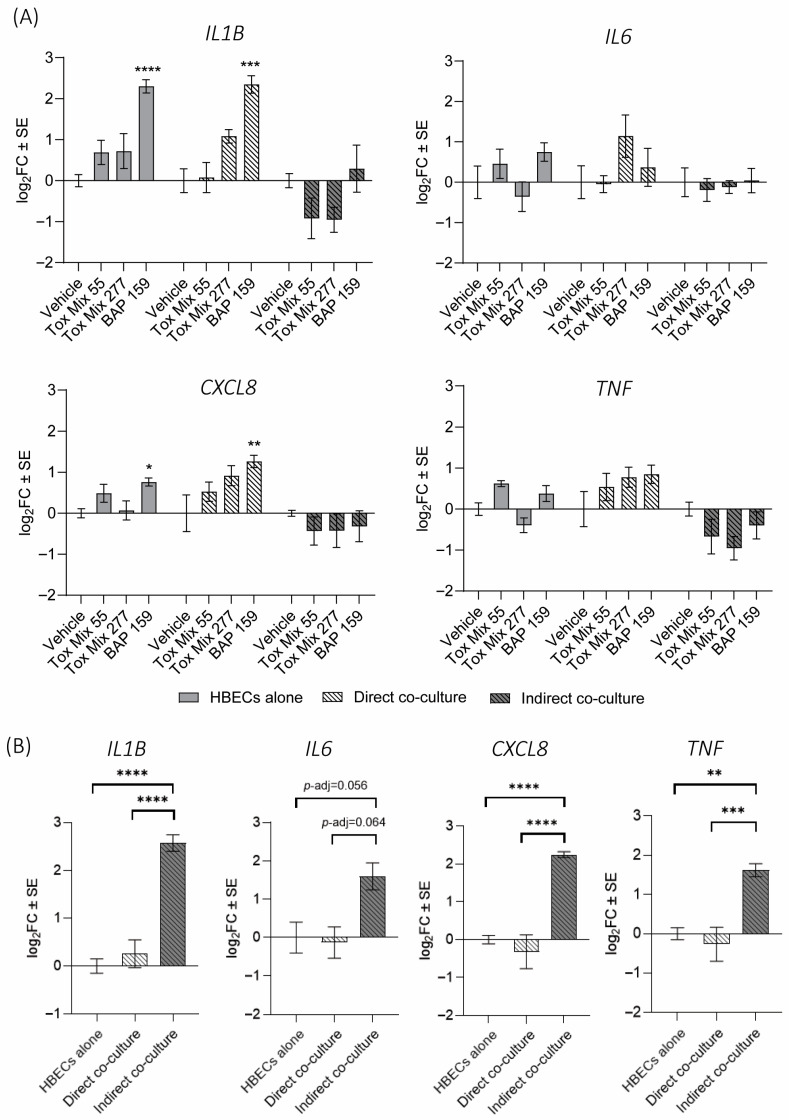
Cytokine gene expression in HBECs. HBECs alone and in direct and indirect co-cultures were exposed to vehicle, Tox Mix, or BAP in 1% DMSO vehicle control. HBECs were then isolated and evaluated for *IL1B*, *IL6*, *CXCL8*, and *TNF* gene expression. For clearer visualization of treatment effects, (**A**) shows the data normalized to the respective model vehicle, and (**B**) shows the vehicle groups normalized to the HBEC-alone vehicle. Data points represent the mean log_2_ fold change (log_2_FC) in gene expression compared to either the respective model vehicle (**A**) or the HBEC-alone vehicle (**B**). Error bars represent the standard error of the means. Statistical significance is indicated by asterisks (* *p*-adj < 0.05, ** *p*-adj < 0.01, *** *p*-adj < 0.001, **** *p*-adj < 0.0001; two-way ANOVA followed by Tukey’s post hoc test). Nearly statistically significant responses are labeled with their adjusted *p*-values from Tukey’s test.

Overall, only *IL1B* and *CXCL8* were significantly increased (*p* < 0.05) after treatment, which was observed in both a model and treatment-specific manner (Figure 4). Specifically, HBEC cultured alone and from the direct co-culture model had significantly elevated *IL1B* and *CXCL8* after BAP treatment with cells from the direct co-culture expressing similar or greater magnitude of cytokine response compared to HBEC cultured alone (Figure 4A). For *IL1B*, the treatment effect, model effect, and interaction between model and treatment were all statistically significant by two-way ANOVA (*p* < 0.0001, *p* < 0.0001, *p* = 0.0269, respectively, Figure 4A). For *CXCL8*, there was a statistically significant difference by model (*p* < 0.0001) and moderate treatment effect (*p* = 0.0648). While *IL6* and *TNF* expression were not significantly changed with treatment after 24 h, there was a trend toward increased expression in direct co-culture.

In contrast, HBEC in indirect co-culture generally appeared to be the least sensitive to changes in cytokine gene expression after chemical treatment (Figure 4A). This may be due to already elevated cytokine expression levels in HBEC in indirect co-culture compared to the other two models after vehicle treatment (Figure 4B). This elevated cytokine response in the indirect co-culture control may make the model less sensitive to responding to subsequent chemical exposures and limits its usefulness for PAH toxicity testing. Neither Tox Mix nor BAP resulted in statistically significant differential expression of these cytokines compared to vehicle in HBEC in indirect co-culture, and most showed a trend toward decreased expression with treatment compared to control (Figure 4A).

### 3.5. PAH Biomarker Expression in HBEC

Expression of *CYP1A1*, *CYP1B1*, *ALDH3A1*, *HMOX1*, *NQO1*, and *TJP2* mRNA was evaluated in HBECs as typical biomarkers of PAH exposure and toxicity associated with chemical metabolism, oxidative stress, and barrier integrity [30,55]. *CYP1A1* and *CYP1B1* encode the eponymous phase I enzymes that contribute to production of carcinogenic metabolites from PAHs such as benzo[a]pyrene-7,8-dihydrodiol-9,10-epoxide (BPDE) from BAP. *CYP1A1* and *CYP1B1* are regulated by the aryl hydrocarbon receptor (AHR), which PAHs can bind to and induce *CYP1A1* and *CYP1B1* expression among other genes [30]. *ALDH3A1*, *HMOX1*, and *NQO1* encode genes that are differentially expressed in response to oxidative stress, with *ALDH3A1* being associated with reactive aldehydes and *HMOX1* and *NQO1* associated with reactive oxygen species [61,62]. Like *CYP1A1* and *CYP1B1*, *ALDH3A1* induction can also be mediated by the AHR in response to xenobiotics [61]. Lastly, *TJP2* encodes a tight junction protein that plays a role in the formation and function of tight junctions between epithelial cells [63].

**Figure 5:**
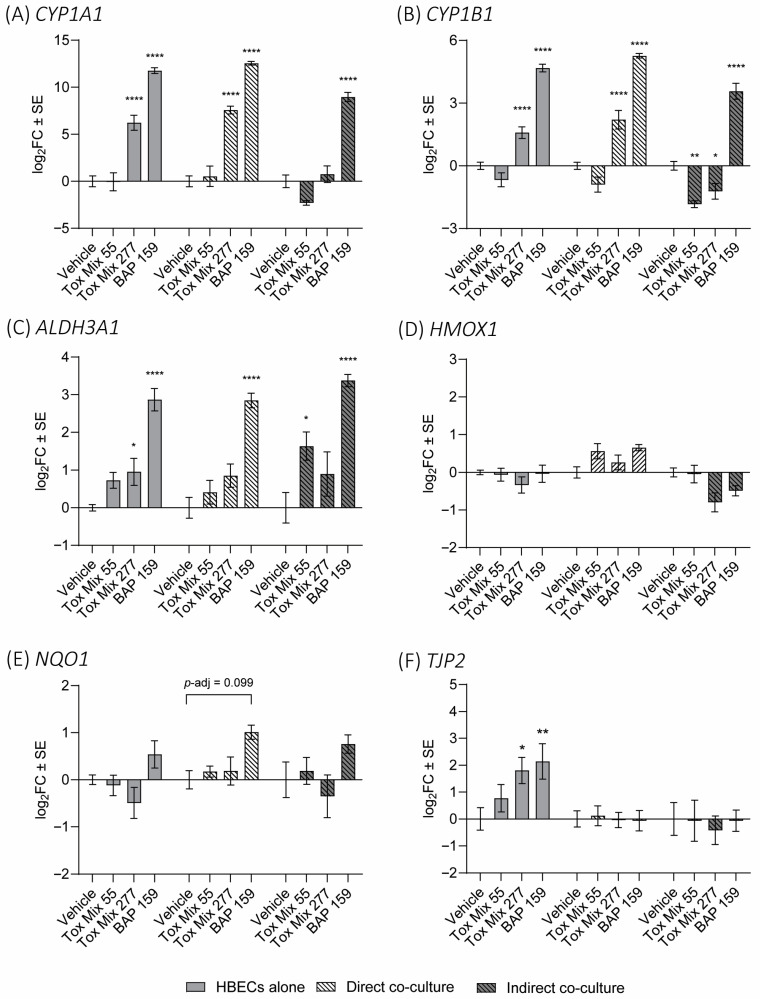
Gene expression in HBEC after treatment with PAHs. HBECs alone and in direct and indirect co-cultures were exposed to vehicle, Tox Mix, or BAP in 1% DMSO vehicle control. HBECs were then isolated and evaluated for (**A**) *CYP1A1*, (**B**) *CYP1B1*, (**C**) *ALDH3A1*, (**D**) *HMOX1*, (**E**) *NQO1*, and (**F**) *TJP2* expression. Data points represent the mean log_2_ fold change (log_2_FC) in gene expression compared to either of the respective model vehicles. Error bars represent the standard error of the means. Statistical significance is indicated by asterisks (* *p*-adj < 0.05, ** *p*-adj < 0.01, **** *p*-adj < 0.0001; two-way ANOVA followed by Tukey’s post hoc test).

For *CYP1A1* and *CYP1B1*, the model effect, treatment effect, and interaction between these two were all statistically significant by two-way ANOVA (*p* < 0.0001, *p* < 0.0001, and *p* ≤ 0.0003, respectively). There was significant induction of *CYP1A1* and *CYP1B1* following BAP treatment in all models compared to vehicle (*p*-adj < 0.0001; Figure 5A,B) and significant induction following 277 µM Tox Mix treatment in HBECs alone and the direct co-culture (*p*-adj < 0.0001). Similarly to cytokine response, cells from the direct co-culture expressed a similar or greater magnitude of CYP450 expression compared to HBECs cultured alone. In contrast, HBECs from the indirect co-culture exhibited a lower magnitude of *CYP1A1* and *CYP1B1* expression compared to control cells with Tox Mix resulting in significant reductions in *CYP1B1* expression (*p*-adj < 0.01 and *p*-adj < 0.05 for 55 and 277 µM, respectively). Of the treatments, BAP resulted in the greatest induction of *CYP1A1* and *CYP1B1* mRNA across models. Overall, HBECs in direct co-culture were more sensitive in changes in CYP expression compared to HBECs in indirect co-culture and showed a similar response to HBECs alone. Notably, differences between co-culture models were most apparent in the Tox Mix treatment groups. A non-monotonic trend in *CYP1B1* expression was observed in HBECs alone and direct co-culture after Tox Mix treatment. Previously, *CYP1B1* has shown this non-monotonic relationship in HBECs alone, with low doses of PAHs slightly reducing expression and higher doses drastically inducing expression [26,32,55].

The oxidative stress marker *ALDH3A1* appeared to show the most consistent response across the models, and consequently, there was no significant model or interaction effect by two-way ANOVA (*p* = 0.1734 and *p* = 0.5949, respectively; Figure 5C). However, there was a significant treatment effect (*p* < 0.0001). For all three models, there was a significant induction of *ALDH3A1* following BAP treatment compared to the vehicle (*p*-adj < 0.0001 for all), and all three models showed induction following Tox Mix treatment despite limited statistical significance.

The other oxidative stress markers, *HMOX1* and *NQO1*, did not result in any significant changes in expression after treatment across model types; however, there was a significant model effect observed for *HMOX1* by two-way ANOVA (*p* < 0.0001; Figure 5D) in which expression after PAH treatment was generally increased in the direct co-culture model and decreased in the indirect co-culture. For *NQO1*, only the treatment effect was statistically significant by two-way ANOVA (*p* = 0.0008; Figure 5E), with BAP showing a trend in increased expression of *NQO1* across all models.

Finally, for *TJP2*, a component of tight junction barrier integrity, there was a significant model effect (*p* = 0.0010; Figure 5F) in which only HBECs cultured alone exhibited significant induction of *TJP2* following 277 µM Tox Mix and BAP treatment by Tukey’s post hoc test (*p*-adj < 0.05 and *p*-adj < 0.01, respectively) compared to either co-culture model. In a previous study using HBECs alone, *CYP1A1*, *CYP1B1*, *ALDH3A1*, *HMOX1*, *NQO1*, and *TJP2* responses were evaluated following Tox Mix treatment and were similar to the results of this study [55]. For BAP, another study also showed increases in *CYP1A1* and *CYP1B1* after a 48 h treatment; however, no statistically significant change in *TJP2* was observed from BAP treatment in HBECs alone, which suggests a time-dependent response in *TJP2* [26]. Absent of PAH treatment, differences in constitutive expression of the PAH biomarkers were observed between models (Supplemental Figure S2), although changes in magnitude of response were less than 2-fold for all biomarkers except *TJP2*. *TJP2* expression was significantly increased in the indirect co-culture (mean of 3-fold log_2_FC) compared to the HBECs alone and direct co-culture.

In studies using intestinal co-culture models, Tamura et al. (2025) observed a decrease in barrier integrity in C2BBe1 intestinal epithelial cells when RAW264 macrophages were added to the system [64]. Additionally, Spalinger et al. (2020) evaluated the effect of THP-1 macrophages, which were activated to various states, on barrier integrity of HT-29.cl19a/Caco-2BBe cells and found that M0 and M2 macrophages resulted in increased barrier integrity, whereas M1 macrophages and monocytes resulted in decreased barrier integrity [65]. Furthermore, they identified a protein, TCPTP, and its associated gene, *PTPN2*, that may play a role in maintaining barrier integrity in the intestinal cells. Partially based on intestinal research, Ghisalberti et al. (2016) proposed the use of spermidine, a TCPTP agonist, to restore barrier integrity in lung disease patients [66]. The role of *PTPN2* in maintaining the barrier of nasal epithelial cells was investigated by Zhang et al. (2023) [67]. They proposed that hypoxia induces HIF-1α and suppresses *PTPN2* expression. While mechanisms other than HIF-1α mediation are possible, it is worth noting that certain pro-inflammatory factors (TNF and IL-4) have been shown to induce HIF-1α in BEAS-2B cells under normoxic conditions [68]. TNF/IL-4 treatment alone induced HIF-1α to similar levels as hypoxic conditions at 18 and 24 h. Therefore, *TJP2* expression may be modulated by induction of HIF-1α by signaling molecules from macrophages in co-culture, and future work investigating the role of epithelial–macrophage crosstalk in modulating the epithelial barrier could investigate this further.

## 4. Conclusions

The present study describes the development of a primary HBEC and macrophage co-culture organotypic model to investigate the combined contributions of each cell type to PAH toxicity in vitro. Primary HBECs differentiated at the ALI were employed, retaining key features of the in vivo airway epithelium. In contrast to spherical organoid models, primary HBECs grown on cell culture inserts can differentiate at the ALI, which produces in vivo-like characteristics such as diverse epithelial cell types (ciliated and goblet cells, etc.), tight junction-mediated barrier formation, and a pseudostratified architecture [18,26,27,69]. Organotypic HBEC models more closely replicate in vivo transcriptional profiles than undifferentiated or immortalized cell lines, making Transwell^®^ ALI systems well-suited for mechanistic evaluations of chemicals and mixture toxicity [25]. Two different co-culture formats were established to optimize and compare ease of use with potential for communication between cell types in response to PAH toxicity. In the direct co-culture model, macrophages were seeded onto the underside of the Transwell^®^ insert, whereas in the indirect model, macrophages were cultured on the bottom of the well. Both co-culture systems, along with HBECs alone, were exposed to benzo[a]pyrene and a synthetic PAH mixture for assessment of pro-inflammatory cytokine expression, biomarkers of PAH exposure, cytotoxicity, and barrier integrity. HBECs in the indirect co-culture model were generally less responsive to PAH exposure than those in the direct co-culture model, as indicated by lower cytokine transcript levels and reduced PAH biomarker expression. The direct co-culture demonstrated increased inflammatory cytokine gene expression and increased sensitivity in *CYP1A1* and *CYP1B1* induction compared to the indirect co-culture. These findings suggest that the direct co-culture model may be more suitable for assessing PAH toxicity, whereas the indirect model demonstrated decreased sensitivity to PAH mixture exposure. Additionally, interactions between model type and treatment were observed, indicating that both macrophages or steps in model setup play significant roles in modulating responses to PAHs. Overall, these findings demonstrate that co-culture configuration can substantially impact response to PAH exposure for certain endpoints and should be carefully considered when evaluating toxicity in physiologically relevant in vitro systems. Future in vitro studies may benefit from exploring a similar comparison of responses between models to chemicals of other classes since this work was focused on PAHs.

## Data Availability

The original contributions presented in this study are included in the article/Supplementary Materials. Further inquiries can be directed to the corresponding author.

## Supplementary Materials

The following supporting information can be downloaded at https://www.mdpi.com/article/10.3390/toxics13121065/s1. Figure S1: Overview of co-culture models; Figure S2: Gene expression in HBEC vehicle controls; Table S1: Chemical Standards; Table S2: Primer sequences. References [10,23,48,70,71] are cited in Supplementary Materials.
