# Supplementary material for: Co-Culture of Primary Human Bronchial Epithelial Cells at the Air–Liquid Interface and THP-1 Macrophages to Investigate the Toxicity of Polycyclic Aromatic Hydrocarbons
Source: Toxics. 2025 Dec 9;13(12):1065. doi: 10.3390/toxics13121065 (PMC12737494; doi:10.3390/toxics13121065)
Supplement: Supplementary file 1 [file toxics-13-01065-s001.zip › toxics-4010762-supplementary.pdf]

Co-culture of primary human bronchial epithelial cells at the air-liquid interface and THP-1 macrophages to investigate the toxicity of polycyclic aromatic hydrocarbons

Kyle S. Burns 1,2, Audrey G. Biggerstaff 1, Jamie M. Pennington 1 and Susan C. Tilton 1,2,*

1 Department of Environmental and Molecular Toxicology, Oregon State University,

Corvallis, OR 97331, USA; kyle.burns@oregonstate.edu (K.S.B.); biggerau@oregonstate.edu (A.G.B.);

jamie.pennington@oregonstate.edu (J.M.P.)

2 Superfund Research Center, Oregon State University, Corvallis, OR 97331, USA

* Correspondence: susan.tilton@oregonstate.edu

Supplemental Tables and Figures

Table S1. Chemical Standards.

Chemical	% of Mixture by molar amount	CAS #	Purity	Supplier
retene	72.8	483-65-8	97%	Santa Cruz Biotechnology
benzo[a]fluorene	14.6	238-84-6	>98%	Santa Cruz Biotechnology
benzo[b]fluorene	7.28	243-17-4	98.1%	AccuStandard
benzo[c]fluorene	3.64	205-12-9	98%	TRC
triphenylene	1.45	217-59-4	99.4%	AccuStandard
benzo[e]pyrene	0.145	192-97-2	99.9%	AccuStandard
benzo[ghi]perylene	0.091	191-24-2	98.9%	AccuStandard

Table S2. Primer sequences.

Gene	Forward (F)/ Reverse (R)	Primer sequence 5'-3'	NCBI Gene ID	Reference
PPIA	F	GCATACGGGTCCTGGCATCTTGTCC	5478	Chang et al., 2019
PPIA	R	ATGGTGATCTTCTTGCTGGTCTTGC	5478	Chang et al., 2019
IL1B	F	ATGATGGCTTATTACAGTGGCAA	3553	Wang et al., 2020
IL1B	R	GTCGGAGATTCGTAGCTGGA	3553	Wang et al., 2020
IL6	F	ACTCACCTCTTCAGAACGAATTG	3569	Wang et al., 2020
IL6	R	CCATCTTTGGAAGGTTCAGGTTG	3569	Wang et al., 2020
CXCL8	F	ACTGAGAGTGATTGAGAGTGGAC	3576	Wang et al., 2020
CXCL8	R	AACCCTCTGCACCCAGTTTTC	3576	Wang et al., 2020
TNFTNF	F	GAGGCCAAGCCCTGGTATG	7124	Wang et al., 2020
TNFTNF	R	CGGGCCGATTGATCTCAGC	7124	Wang et al., 2020
ALDH3A1	F	TGTTCTCCAGCAACGACAAGG	218	Chang et al., 2019
ALDH3A1	R	AGGGCAGAGAGTGCAAGGT	218	Chang et al., 2019
HMOX1	F	CTCTGAAGTTTAGGCCATTG	3162	Thakor et al., 2011
HMOX1	R	AGTTGCTGTAGGGCTTTATG	3162	Thakor et al., 2011
NQO1	F	GAAGAGCACTGATCGTACTGGC	1728	Chang et al., 2019
NQO1	R	GGATACTGAAAGTTCGCAGGG	1728	Chang et al., 2019
TJP2	F	GGCCTACGACCCAGACTAC	9414	Chang et al., 2019
TJP2	R	ACTCTTCGTTCGCTCTGCTTT	9414	Chang et al., 2019
ACTB	F	ATTGCCGACAGGATGCAGAA	60	MaeB et al., 2014
ACTB	R	GCTGATCCACATCTGCTGGAA	60	MaeB et al., 2014
CYP1A1	F	TCGGCCACGGAGTTTCTTC	1543	Chang et al., 2019
CYP1A1	R	GGTCAGCATGTGCCCAATCA	1543	Chang et al., 2019
CYP1B1	F	CCAACCTGCCCTATGTCCT	1545	Chang et al., 2019
CYP1B1	R	CTGGATCAAAGTTCTCCGGG	1545	Chang et al., 2019
CD14	F	CTGGAACAGGTGCCTAAAGGAC	929	Betancur et al., 2022
CD14	R	GTCCAGTGTCAGGTTATCCACC	929	Betancur et al., 2022

Supplemental Figure S1.

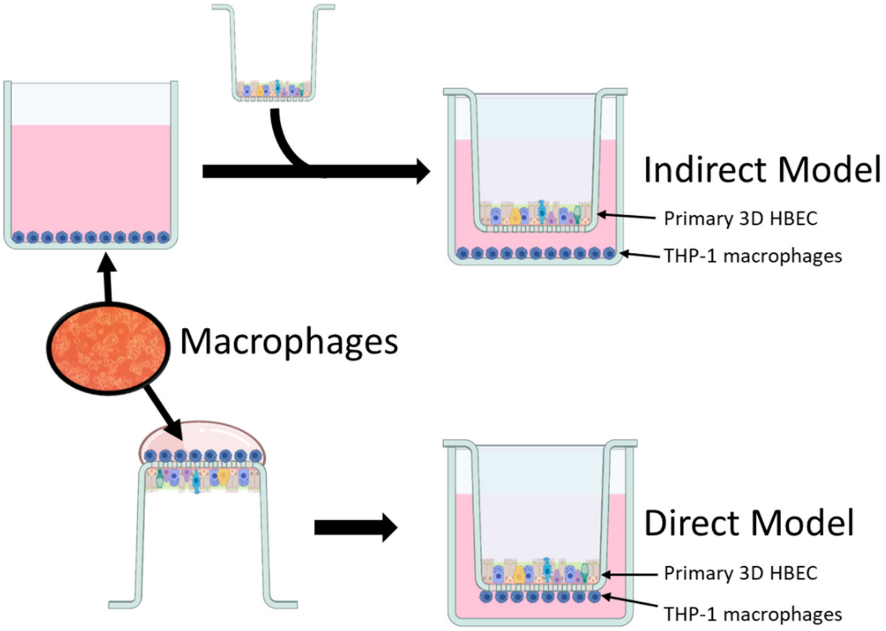

Supplemental Figure S1. Overview of co-culture models. Two co-culture methods were utilized in this study and are referred to as the indirect co-culture model and the direct co-culture model. In the indirect co-culture model (top), THP-1 macrophages were seeded onto the bottom of a cell culture plate well. In the direct co-culture model (bottom), THP-1 macrophages were seeded onto the inverted Transwell® insert. Both models utilized primary HBECs, containing a variety of epithelial subtypes, on the apical side of the Transwell® insert and THP-1 macrophages in the basolateral chamber. HBEC and THP-1 cells were cultured on opposite sides of the Transwell® membrane to simplify analysis and determination of how each cell type (HBEC or macrophage) is contributing to the response to PAH exposure.

Supplemental Figure S2.

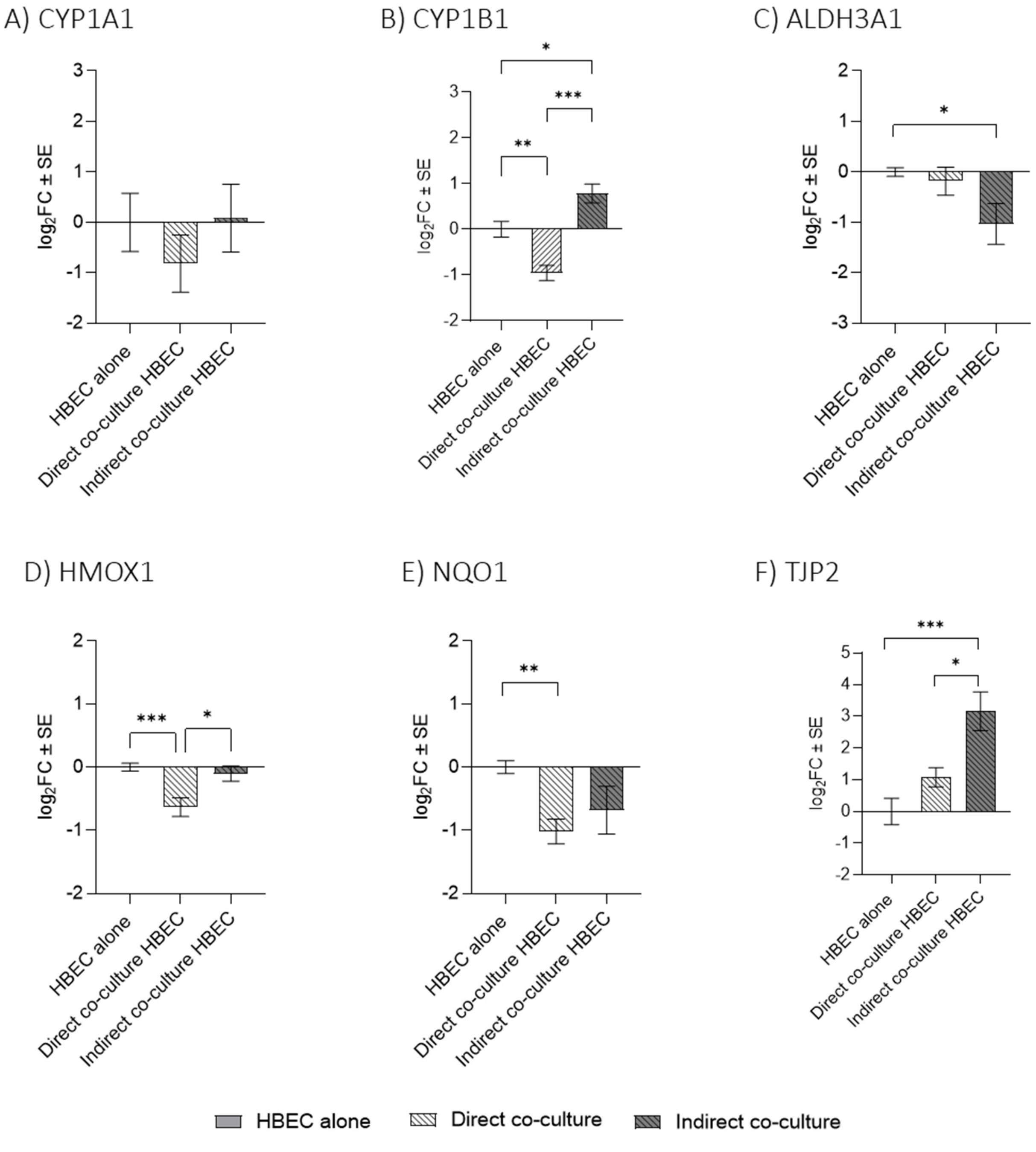

Supplemental Figure S2. Gene expression in HBEC vehicle controls. HBEC alone and in direct and indirect co-cultures were exposed to in 1% DMSO vehicle control and then isolated and evaluated for (A) *CYP1A1,* (B) *CYP1B1,* (C) *ALDH3A1*, (D) *HMOX1*, (E) *NQO1*, and (F) *TJP2* expression. Data points represent the mean response in each platform

normalized to HBEC alone vehicle and presented as the mean log_2 fold change (log_2FC) normalized to HBEC alone. Error bars represent the standard error (SE) of the means. Statistical significance is indicated by asterisks (*p-adj<0.05, **p-adj<0.01, ***p-adj<0.001, ****p-adj<0.0001; One-way ANOVA followed by Tukey’s post-hoc test).
